# Exercise in cancer patients: assistance levels and referral pathways—a position statement from the Spanish Society of Medical Oncology

**DOI:** 10.1007/s12094-024-03546-w

**Published:** 2024-06-23

**Authors:** Blanca Herrero López, Ana Cardeña-Gutiérrez, Ana Godoy Ortiz, Ana Gonzaga López, Ana María Grueso López, Ana Nuño Alves, Patricia Ramírez Daffós, César A. Rodríguez Sánchez, Ángel R. Rodríguez Pérez, Víctor Sacristán Santos, Salvador Saura Grau, Raquel Sebio García, Miguel Ángel Seguí Palmer

**Affiliations:** 1 Hospital General Universitario Gregorio Marañón, Instituto de Investigación Sanitaria Gregorio Marañón. Madrid (España), Madrid, Spain; 2 Hospital Universitario Nuestra Señora de Candelaria. Santa Cruz de Tenerife (España), Santa Cruz de Tenerife, Spain; 3 UGCI Oncología Médica Hospitales Universitarios Regional y Virgen de La Victoria, Málaga, Málaga, Spain; 4 Hospital de Sagunto. Valencia (España), Port de Sagunt, Spain; 5 Hospital Universitario Virgen Macarena, Seville, Spain; 6 Hospital Obispo Polanco, Teruel, Spain; 7 Hospital Puerta del Mar, Cádiz, Spain; 8 Hospital Clínico Universitario de Salamanca-IBSAL, Salamanca, Spain; 9 Hospital Universitario Fundación Jiménez Díaz, Madrid, Spain; 10 Complejo Hospitalario Universitario A Coruña, A Coruña, Spain; 11 Hospital Universitario de Gran Canaria Dr. Negrín, Las Palmas de Gran Canaria, Spain; 12 Servicio de Rehabilitación. Hospital Clínic de Barcelona, Barcelona, Spain; 13 Parc Taulí Consorci Corporació Sanitaria. Sabadell. Servicio de Oncologia. Institut d’Investigació I Innovació Parc Taulí, Universitat Autònoma de Barcelona, Barcelona, Spain

**Keywords:** Cancer, Cancer care, Exercise, Exercise therapy, Integrative care in oncology, Multidisciplinary, Multidisciplinary approach, Oncology, Supportive care, Treatment

## Abstract

There is growing evidence about how physical activity can improve cancer care. Unfortunately, exercise is still not widely prescribed to oncology patients, despite the benefit it brings. For this to occur, it is necessary for a multidisciplinary approach involving different types of healthcare professionals, given that each treatment be tailored for each single case. Besides incorporating appropriate infrastructures and referral pathways, we need to integrate exercise into healthcare practice, which ameliorates patients’ quality of life and treatment side effects. From the Spanish Society of Medical Oncology (SEOM), and through the Exercise and Cancer Working Group, we indicate considerations, analyze patient care scenarios, and propose a referral pathway algorithm for exercise prescription, taking in account the patient’s needs. In later sections of this paper, we describe how this algorithm could be implemented, and how the exercise programs should be built, including the physical activity contents, the settings, and the delivery mode. We conclude that professionals, infrastructures, and organizations should be available at every assistance level to create programs providing adequate exercise training for cancer patients.

## Introduction

Modern cancer care requires a multidisciplinary approach for each patient. The number of cancer survivors is constantly increasing, thanks to the recent advances in diagnosis and treatment; approximately two-thirds of cancer patients will reach long-term survival for over 5 years [1, 2]. About 25% of the world’s adult population does not meet world health organization (WHO) physical activity recommendations. However, exercise is an essential element in maintaining a healthy lifestyle. It is consistently related to lower incidence and mortality rates of several cancer types [3, 4]. Moreover, regular exercise practice is associated with improvements in quality of life (QoL) and fewer treatment-related side effects [5].

Evidence shows that exercise can reduce up to 30% the risk of breast, colon, bladder, endometrial, esophageal, and gastric cancers [6]. As well, it is also associated with an almost 20% risk reduction of disease-specific mortality for all cancers combined. The largest and better-designed studies of survival outcomes focus on breast and colorectal cancer, with clear reductions in cancer-specific and overall mortality. Interestingly, it also shows improvements in mortality outcomes for other cancer locations such as prostate or lung [3].

Cancer diagnosis and antineoplastic treatments imply a cardiovascular fitness worsening. Both lead to body composition changes, multiple treatment-derived adverse effects, e.g., nausea, diarrhea, insomnia, fatigue, etc., and, ultimately, a global quality of life deterioration. Aerobic and resistance regular exercise should be recommended for patients undergoing cancer treatment, given that it is related to cardiorespiratory fitness enhancement, along with fatigue and patient-reported outcomes (PROs) improvements [7].

The biological mechanisms whereby exercise interferes with cancer development and progression target all hallmarks of cancer. It reduces anabolic and sexual hormones’ production, regulates apoptosis-related gene expression, favoring cancer cell death via myokines production, helps to restrain cell-cycle control, increases tissue perfusion and vascularization, interferes with cancer cell invasion by enhancing adhesion protein expression, and improves immune system function [6, 8, 9].

Despite the plethora of scientific evidence about the benefits before and after cancer diagnosis and treatment, very few patients remain physically active. Multiple factors may influence this: a lack of training for medical doctors and other healthcare professionals, that should be able to identify cancer patients who can practice exercise without supervision, and those who need specific attention and monitoring; a need for more research to better understand each patient’s biological and physiological changes with exercise, to identify the adequate exercise intensity and frequency that should be prescribed; and a lack of enough multidisciplinary units or programs dedicated to exercise in oncology in health care centers. In turn, patient status may influence decision-making on exercise practice: the presence of disease or treatment-related symptoms, e.g., pain or nausea, limited self-confidence, lack of motivation, fear of potential exercise-induced adverse effects, or different socioeconomic difficulties to gain access to exercise services [4, 10].

In this position statement, the Spanish Society of Medical Oncology (SEOM) through the Exercise and Cancer Working Group aims to generate and provide practical evidence-based recommendations for clinicians and to optimize exercise practice for cancer patients and refer them to the adequate setting.

## General considerations for exercise prescription in *cancer* patients

Exercise prescription in clinical practice requires performing a complete pre-exercise assessment. The objectives are to identify potential risks and barriers of exercise practice and to achieve the greatest benefit for each patient and clinical situation. Some of these barriers make the screening process difficult for oncology care providers, such as limited available time during medical appointments, lack of training on exercise prescription, and lack of human and economic resources [11, 12].

**Fig. 1:**
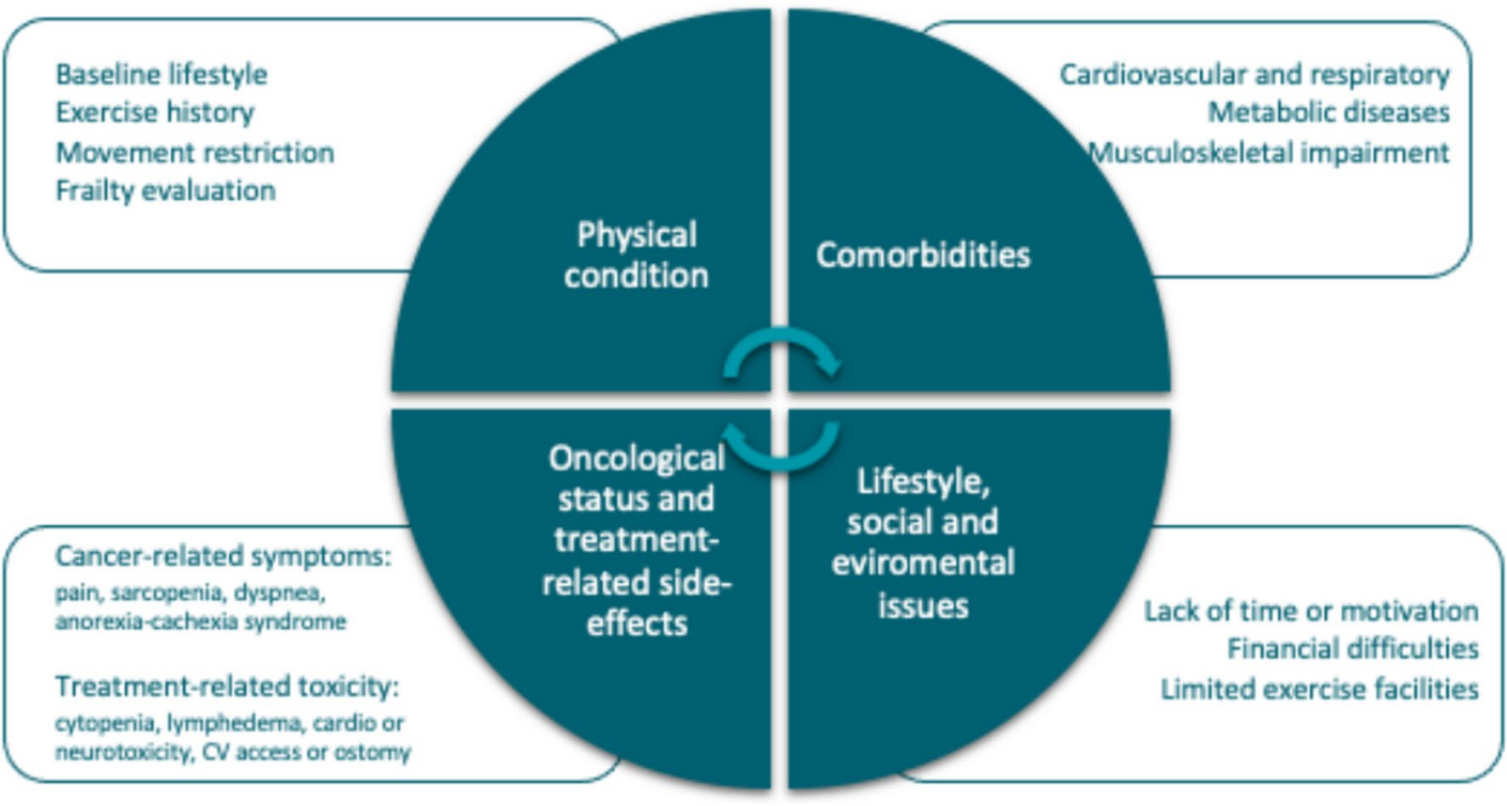
Factors to consider for exercise prescription in cancer patients

A comprehensive approach including physical, emotional, and behavioral state of the patient may help oncology care providers to recognize the main issues influencing personalized exercise prescription. Several factors need to be considered before exercise prescription for cancer patients [11–13]. They are summarized in the next four sections (Fig. 1).

### Physical condition evaluation

Determination of a baseline lifestyle—active or sedentary—previous exercise history, movement restriction, and level of physical activity knowledge can help to establish the starting point for exercise prescription. Some validated scales may also be useful to address baseline physical activity such as the Godin–Shepard leisure-time exercise questionnaire [14] or the international physical activity questionnaire (IPAQ) [15].

It is essential to identify age-related physical conditions, e.g., fall risk, cognitive impairment, mobility issues, social issues, geriatric syndromes, that may have an impact on exercise prescription. In this setting, applying elderly frailty scales for screening such as the G8 screening tool [16] or the Yale Physical Activity Survey scale is recommended [16].

### Comorbidities assessment

Cancer patients usually exhibit other cardiovascular, respiratory, and metabolic diseases, as well as articular or musculoskeletal impairments. Those might limit their exercise capacity such as hypertension, arrhythmia, history of ischemic heart disease, deep-vein thrombosis, pulmonary embolism, etc. Furthermore, patients with previous or ongoing anthracycline-based treatment, anti-HER2 therapies, tyrosine kinase inhibitors or previous thoracic radiotherapy (RT) may develop treatment-related cardiotoxicity. Respiratory diseases are also common diagnoses in cancer patients—chronic obstructive pulmonary disease (COPD), smoking habits or pneumonitis.

These and other health conditions may have an impact on exercise type and intensity: recommendations can be adapted for patients with musculoskeletal impairment, such as arthralgia, arthrosis, arthritis, movement limitation, or metabolic diseases, such as type 1 or 2 diabetes mellitus, hyperlipidemia, obesity, malnutrition. An initial and regular checkout of relevant patient comorbidities is recommended. This approach would be helpful to customize exercise recommendations and reduce potential risks.

### Oncological status and treatment-related side effects

Oncology care providers and cancer patients may be concerned about the safety of practicing physical exercise. However, it has been shown that exercise is safe during all the cancer care continuum, even during cancer treatment [17]. Regular exercise is associated with decreased treatment’s toxicity and, potentially, response rate improvements [18, 19].

Exercise prescription for cancer patients requires weighting treatment-related toxicities as well as cancer stage and location: patients may present mobility limitations due to brain metastasis, increased risk of fracture or pain related to bone metastasis, surgery sequelae such as lymphedema, ostomies, painful scars, and other special situations derived from peripheral or central venous carriers. Another important issue is the potential risk of cytopenia; in these cases, exercise protocols may need to be modified [20].

### Lifestyle, social, and environmental issues

Understanding the patient’s daily leisure time availability, being an active worker or retired, as well as social and family support is required before exercise prescription. Environmental factors such as exercise place (outdoor or indoor), location (rural or city), home-based or gym-based programs and the presence of exercise facilities also must be taken into account. Furthermore, lifestyle habits like diet and related variables such as weight have an important impact on exercise prescription. Financial difficulties, frequently derived from cancer diagnosis and treatments, are usually underestimated and must be considered along with educational level when prescribing exercise.

Cancer patients require oncology care providers to keep in mind all these elements to bring them an adequate and safe exercise prescription [13]. We recommend a regular assessment of treatment status, exercise capacity, comorbidities and relevant treatment or disease-related symptoms.

## *Cancer* patients’ scenarios for exercise training

Regular physical activity and structured exercise can be delivered in a multiphasic way throughout the disease. Most of the current evidence focuses on the role of physical activity and exercise training for cancer survivors after active treatment is finished. However, during the last few years, there is a growing body of evidence focusing on patients’ engagement in exercise programs across all cancer phases: before treatment, during treatment, after treatment, and in palliative care settings.

### Prehabilitation

Prehabilitation is the period between a major diagnosis, i.e., cancer, and the beginning of targeted therapies, i.e., surgery or chemotherapy (CT) [21]. Multimodal prehabilitation including exercise training, nutritional optimization/ supplementation, and psychological support is usually delivered when tumor resection is the first line of treatment, given that a few weeks are available before surgery. In a recent international multi-center randomized controlled trial, 4 weeks of supervised multimodal prehabilitation including exercise training decrease postoperative complications and enhance recovery 4 weeks after surgery [22]. Importantly, as some patients will undergo adjuvant therapy after surgery, recovery of functional capacity should be a key outcome of prehabilitation.

Unfortunately, prehabilitation and particularly exercise training before systemic cancer treatment have received much less attention, given the short window between cancer diagnosis and treatment beginning. However, preliminary evidence shows that exercise training during prehabilitation could lead to an increase in cardiorespiratory fitness (VO_2_max) and muscle function, improving patients’ frail functional status, and making them eligible for treatment [23, 24]. Despite there are no studies that evaluate the effect of preoperative exercise training in patients facing systemic treatment, prehabilitation can also be used throughout the disease continuum, to prepare patients to withstand sequential treatments over time [25].

### Neoadjuvant (NAT) and adjuvant treatment (AT)

NAT and AT refer to the administration of cancer therapies, e.g., CT, radiation therapy (RT), endocrine therapy (ET), before or after a curative therapeutic approach, i.e., surgery, respectively. Exercise is important in NAT/AT settings for several reasons: first, exercise improves surgical outcomes, as well as overall survival and disease-free survival rates [3, 26, 27]; second, exercise has been found to enhance patients’ quality of life and diminish fatigue levels, among other cancer-related symptoms [28, 29]. Overall, incorporating exercise into neoadjuvant treatment can have important benefits for cancer patients [30–32].

### Advanced disease treatment

Due to cancer patient’s heterogeneity, it is necessary to adapt the treatment to each case. As a consequence of the limited scientific evidence of the effectiveness of exercise in this situation, it is important to consult the specialized oncology care team before starting any exercise program during metastatic cancer treatment.

An increasing number of patients undergo not only chemotherapy but also other types of targeted therapy or immunotherapy during their cancer care continuum. Currently, there are no contraindications or specific data for not recommending exercise practice in this population; it is related to quality-of-life and fatigue improvements, bone health maintenance, lower risk of sarcopenia, etc. [33, 34]

RT is also well-established and widely used for treating malignancies. It is generally safe to include exercise in these patients, especially given that RT is usually associated with fewer adverse events compared to systemic treatments [35–38].

### *Cancer* survivors

For cancer survivors, exercise could potentially maintain its benefits by improving physical function, reducing the risk of recurrence, improving depressive symptoms, fatigue and lymphedema, and enhancing overall quality of life [3, 30, 39, 40]. There is not enough data and a weaker level of evidence about cardiac protection and cognitive function impairment [41].

## Referral decision algorithm for exercise training in patients with a *cancer* diagnosis: who, when, and where

**Fig. 2:**
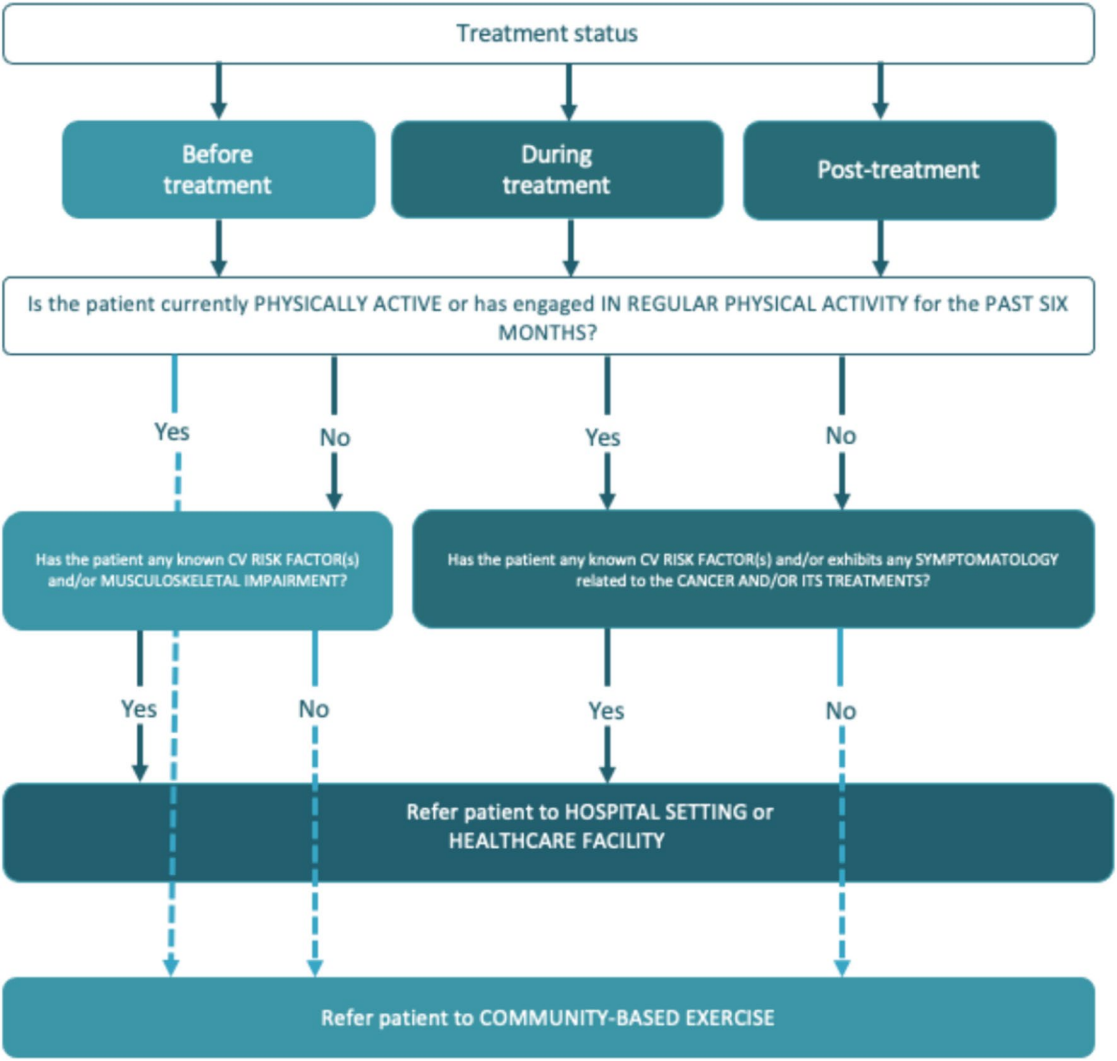
Referral decision algorithm for exercise prescription in cancer patients

Based on the previous sections and considering all the possible situations during the cancer care continuum, we propose different alternatives for patients to engage safely in physical activity and structured exercise. We provide a referral algorithm for oncologists to decide when, where, and who to refer cancer patients for an adequate exercise prescription (see Fig. 2).

## Algorithm implementation and exercise assistance levels

There is dramatically growing evidence supporting the role of exercise programs for people living with and beyond cancer. Unfortunately, implementation of exercise-based programs is still scarce. Several reasons might be responsible for this paucity of programs in clinical practice which include funding and hospital budget policies and priorities, insufficient trained personnel, and lack of well-designed protocols and referral pathways.

To implement an exercise program for cancer patients, three main aspects should be considered: (i) healthcare organization and infrastructure; (ii) human resources and personnel; and (iii) coordination and organization of different strata involved. As a result, availability might vary in both in-hospital and outpatient community-based programs, supplies and staff involved, depending on the local and regional healthcare policies.

### Implementation and coordination of in-hospital exercise programs

Our national healthcare system organization implies that some differences might arise in the distribution of resources and patient–professional ratios, which might hinder the standardization of exercise-based programs in-hospital. Currently, there is wide heterogeneity within our national system in the availability of these programs as well as their complexity and delivery mode. To implement a hospital-based exercise program, it is recommended a structured pathway for patients’ referral, and continuous coordination and communication between the healthcare team. For the latter, an interdisciplinary team encompassing at least a medical oncologist, a physical medicine and rehabilitation doctor, and a physiotherapist is recommended. In addition, other healthcare professionals such as radiation oncologists, oncology nurses, occupational therapists, surgeons, anesthesiologists, and cardiologists could also be included, based on the patients’ needs.

Referral pathways might be difficult to standardize, as they widely vary with each hospital organization and complexity. Most importantly, patients should be referred to the exercise program as soon as possible to minimize the progression of disease-related symptoms and dysfunctions. We recommend that after the clinical assessment by the oncologist, patients should be referred to the exercise program within the following 2 weeks to avoid more deconditioning and prevent further decline. Finally, in terms of material resources, we recommend a hospital gym with some exercise machines or a diaphanous space where directed physical activities can be performed with body weight and/or low-cost equipment, such as elastic bands, free weights, and others.

### Implementation and coordination of community-based exercise programs

Despite in-hospital exercise programs being recommended for close monitoring of patient’s progress, especially for those with more severe limitations or impairments, they cannot be applied indiscriminately, as they usually can only accommodate a selected number of patients. It is important to design specific referral pathways for those patients who can safely exercise outside a healthcare facility. In this case, the interdisciplinary team should be formed at least by the medical oncologist as well as an exercise professional or physiotherapist in the referred facility. In addition, we recommend the inclusion of other healthcare professionals such as oncology nurses, sports medicine doctors or physical medicine and rehabilitation doctors to monitor potential adverse events.

**Fig. 3:**
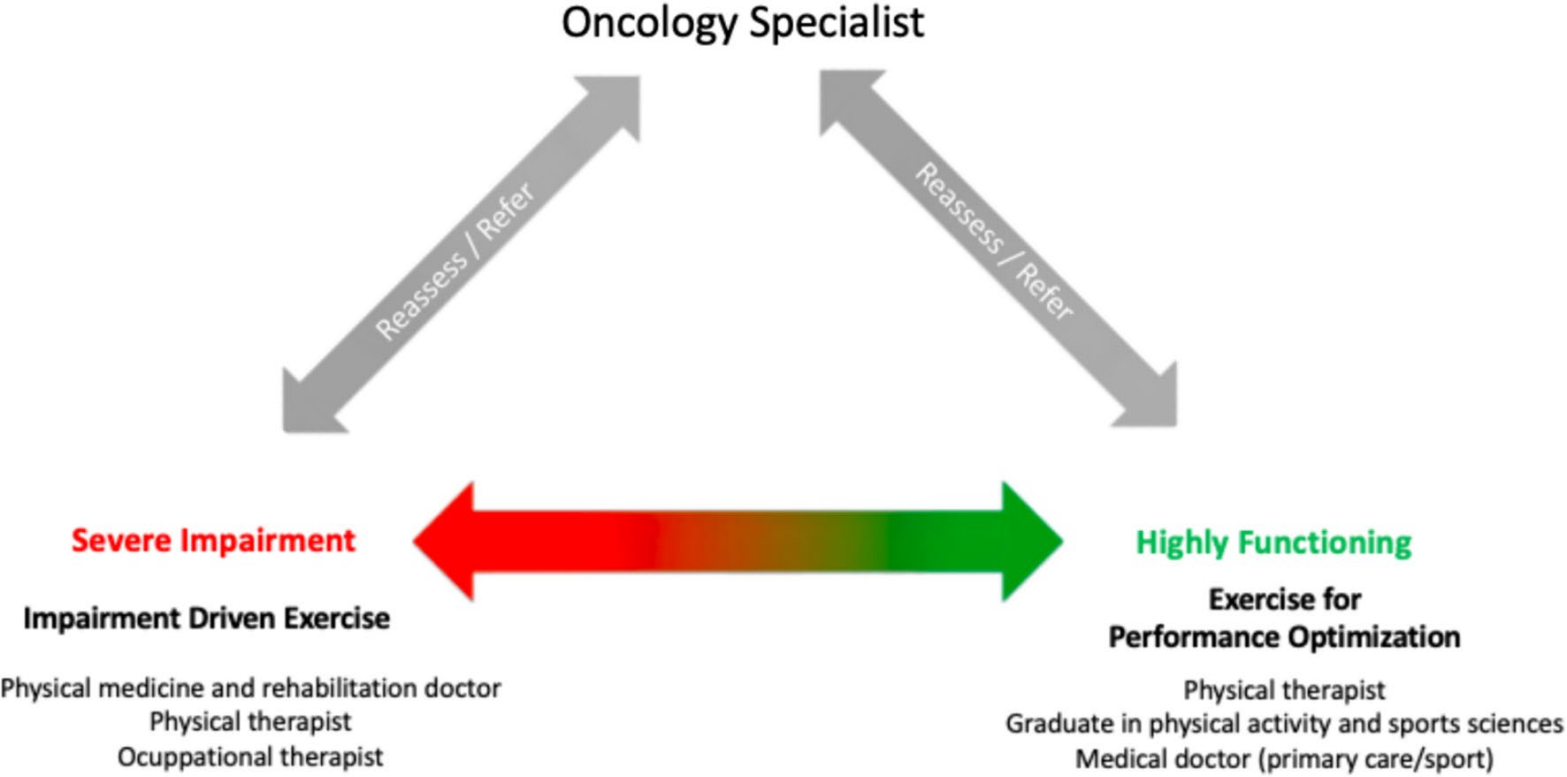
The triangle base represents the functional continuum in cancer patients. Below are the health care or exercise professionals regarding patient complexity. Diagonal arrows represent the oncology specialists’ initial exercise prescription, referral to professional intervention circuits based on the patient's physical capacity or impairments and reassessment recommendations at oncology appointments

Referral pathways for these programs are currently very difficult to define, as they depend on local resources and regional physical activity policies. Ideally, the recommendation for cancer patients about participating in exercise programs should come from medical oncologists, and based on an initial assessment of physical functioning, symptoms, and disease-related impairments; then an exercise professional or experienced physiotherapist could implement it in the community (Fig. 3). Based on the patient’s status and preferences, the professional would choose the most adequate exercise program, as detailed in the following document section.

## Exercise programs

When discussing exercise programs for cancer patients, it is essential to consider the contents, settings, and delivery mode to ensure the safety, effectiveness, and success of planned interventions.

### Contents

An exercise oncology program must combine the best available evidence with the expertise of professionals who apply it. Aerobic and resistance exercises have been the primary focus of most studies on physical activity and cancer [41], while there is less evidence specifically addressing flexibility and balance exercises benefits for cancer patients. All of them are essential components of a comprehensive exercise program.

Aerobic exercises such as walking, jogging, cycling, swimming, and various adaptive training practices improve cardiorespiratory fitness, reduce fatigue, and enhance cancer patient’s quality of life. Resistance exercise training, which involves the use of weights or other forms of resistance to strengthen muscles like elastic bands, constitutes a key component of beneficial physical activity for cancer patients. They help to counteract muscle weakness and lean body mass loss, usually present in cancer patients as treatment-related side effects, improving functional capacity, and reducing fatigue [42]. Flexibility exercises, such as stretching and yoga, help maintain or improve range of motion and reduce muscle stiffness, which is particularly important for patients who may experience limited mobility due to treatment, including surgery. Finally, balance exercises, e.g., tai chi and specific balance training, can help to improve stability and reduce the risk of falls; this exercise modality is of particular importance for cancer patients diagnosed with bone loss or metastases [43].

### Settings

There are many uncertain moments and negativity along the cancer continuum. Understanding patient’s concerns is vital as they often feel vulnerable. For instance, patients may feel intimidated or uncomfortable in places where healthy individuals engage in sports; accordingly, it is important to offer cancer patients a harmonious environment in which they feel physically and psychologically comfortable when practicing exercise [44]. This setting should also help patients familiarize themselves with new materials and exercises through proper instruction. By perceiving emotional needs and creating an empathetic and supportive environment, we can ensure that they feel comfortable and confident while participating in exercise programs [45, 46].

Deciding each appropriate exercise setting for each patient requires to understand the range between high functionality and severe impairment (Fig. 1). For patients with limitations, training goals aim to promote and preserve autonomy and symptomatic control in an in-hospital setting that provides a safe and controlled environment, ensuring the necessary resources and support to address their specific needs [47]. In contrast, for those without significant impairment, exercise practice aims for physical reconditioning and should be carried out on an outpatient basis, with more flexible exercise approaches that allow patients to engage in exercise sessions outside a hospital setting. This scenario fosters a sense of autonomy and empowers them to take an active role in their recovery and health maintenance.

In outpatient exercise settings, supervision can be further divided into professionalized environments and community environments [12]. Professionalized environments include healthcare centers and associations with medical support, that is, healthcare together with physical activity professionals providing specific exercise programs tailored to each patient’s needs [48]. Community environments encompass engagement in activities to raise awareness campaigns within public spaces, fitness centers, or online remote program initiatives led by exercise professionals. These settings offer patients a more accessible way to integrate exercise practice into their daily routines. It helps to also connect with other people facing similar challenges, promoting a sense of community and support.

### Delivery mode

Exercise programs for cancer patients can be conducted through various formats: in-person, online or hybrid, either in group or one-to-one. Each way offers unique benefits and addresses specific patient needs [49].

In-person exercise programs provide direct supervision and guidance from healthcare professionals or certified trainers, immediate feedback, and personalized adjustments to the exercise routine, ensuring a safe and effective exercise practice. Online remotely supported exercise programs offer cancer patients flexibility and convenience, allowing them to participate in physical activity from the comfort of their homes. This format can be particularly beneficial for those with limited mobility or transportation options and can be easier to fit individual schedules, which may also improve adherence and overall program satisfaction [50]. Hybrid exercise programs combine the benefits of both in-person and online formats. These programs typically begin with in-person instruction to ensure proper technique and understanding of the exercises, followed by online sessions for continued support and motivation. This approach allows patients to establish a strong foundation in exercise principles while maintaining the convenience and flexibility of online sessions.

Group exercise sessions, whether conducted in-person or online, offer cancer patients the opportunity to connect and promote the *herd effect*, where patients feel motivated and encouraged by their peers’ progress. Large online group sessions can reach a wider audience, while smaller in-person group sessions may allow for more personalized attention from the instructor and closer connections among participants, further enhancing motivation and adherence. One-on-one exercise sessions, either in-person or online, can be especially beneficial for cancer patients with important limitations, or those requiring closer supervision. These individualized sessions allow healthcare professionals or certified trainers to tailor the interventions specifically to each patient’s condition, ensuring a safe and effective exercise experience. However, one-on-one sessions may be more resource-intensive and costly compared to group sessions, which could limit their availability and accessibility.

## Conclusion

Evidence supporting the benefits of exercise training throughout the cancer continuum is robust and rapidly increasing. However, several factors need to be taken into account to translate effective exercise training programs into routine clinical practice. In this document, the most relevant features regarding exercise program implementation for cancer patients have been highlighted. Given the population heterogeneity, it is imperative to provide different settings which involve a wide variety of healthcare and exercise professionals to ensure an adequate assistance according to the patient’s needs. Professionals, infrastructures, and organizations should be available at all assistance levels to ultimately create programs that provide exercise training for cancer patients.

## Data Availability

This article is a position statement based on the best available scientific evidence at the time, not a research study. We encourage readers to contact us as the Exercise and Cancer Working Group of the Spanish Society of Medical Oncology (SEOM) for further information and guidance.
